# Healthcare Workers and Nonhealthcare Workers Pro-Vaccination Attitude and Its Associated Factors towards COVID-19 Vaccine Globally: A Systematic Review and Meta-Analysis

**DOI:** 10.1155/2022/2443785

**Published:** 2022-10-10

**Authors:** Addisu Dabi Wake

**Affiliations:** Nursing Department, College of Health Sciences, Arsi University, Asella, Ethiopia

## Abstract

**Introduction:**

Coronavirus disease 2019 (COVID-19) pandemic has not been managed and controlled globally. The aim of this systematic review and meta-analysis were to determine the global pro-vaccination attitude and associated factors towards COVID-19 vaccine among healthcare workers (HCWs) and nonhealthcare workers (non-HCWs).

**Methods:**

Different databases such as PubMed, Scopus, EMBASE, and Google Scholar were used. Preferred Reporting Items for Systematic Reviews and Meta-analyses (PRISMA) 2020 flowchart diagram and PRISMA checklist were used for study screening, selection, and inclusion into this systematic review and meta-analysis. Newcastle-Ottawa Scale (NOS) quality assessment criteria for cross-sectional studies were used to assess the included articles.

**Results:**

A total of 51 studies were included into this systematic review and meta-analysis. The meta-analysis revealed that the global pooled prevalence of pro-vaccination attitude towards COVID-19 vaccine among both HCWs and non-HCWs was 61.30% (95%CI: 56.12, 66.47, *I*^2^ = 99.8%: *p*=0.000). Subgroup analysis showed that the global pooled prevalence of pro-vaccination attitude towards COVID-19 vaccine was the lowest (59.77%, 95%CI (51.56, 67.98); *I*^2^ = 99.6%, *p*=0.000) among the HCWs participants and the highest (62.53%, 95%CI (55.39, 69.67); *I*^2^ = 99.8%, *p*=0.000) among the non-HCWs participants and the lowest (54.31%, 95%CI (43, 65.63); *I*^2^ = 99.5%, *p*=0.000) for sample size <700 and the highest (66.49%, 95%CI (60.01, 72.98); *I*^2^ = 99.8%, *p*=0.000) for sample size >700; the lowest (60.70%, 95%CI (54.08, 67.44); *I*^2^ = 93.0%, *p*=0.000) for studies published in 2020 year and the highest (61.31%, 95%CI (55.93, 66.70); *I*^2^ = 99.8%, *p*=0.000) for the studies published after 2020 years. From this systematic review, factors significantly associated with pro-vaccination attitude towards COVID-19 vaccine among HCWs were such as age, gender, race, work experience, home location, having no fear of injections, being a non-smoker, profession, presence of chronic illnesses, allergies, confidence in pharmaceutical companies, history of taking influenza vaccine, vaccine recommendation, perceived risk of new vaccines, perceived utility of vaccine, receiving a seasonal flu vaccination in the last 5 years, working in a private hospital, a high perceived pandemic risk index, low vaccine harm index, high pro-socialness index, being in close contact with a high-risk group, knowledge about the virus, confidence in and expectations about personal protective equipment, and behaviors. The level of positive attitude towards COVID-19 vaccine among non-HCWs ranged from 21.40% to 91.99%. Factors associated with the attitude towards COVID-19 vaccine among non-HCWs were such as age, gender, educational level, occupation, marital status, residency, income, ethnicity, risk for severe course of COVID-19, direct contact with COVID-19 at work, being a health profession, being vaccinated against seasonal flu, perceived benefits, cues to actions, having previous history of vaccination, fear of passing on the disease to relatives, and the year of medical study, studying health-related courses, COVID-19 concern, adherence level to social distancing guidelines, history of chronic disease, being pregnant, perceived vaccine safety, having more information about vaccine effectiveness, mandatory vaccination, being recommended to be vaccinated, lack of confidence in the healthcare system to control epidemic, and belief in COVID-19 vaccines protection from COVID-19 infection.

**Conclusion:**

This meta-analysis revealed that the global estimated pooled prevalence of pro-vaccination attitude towards COVID-19 vaccine among both HCWs and non-HCWs was unsatisfactory. Globally, there is a need for a call for action to cease the crisis of this pandemic.

## 1. Introduction

COVID-19 pandemic has spread quickly over all countries. This pandemic affects all age categories of the population globally [1]. COVID-19 remains as a large burden to the world, and it continues to ravage the world [2]. This pandemic has put a challenge across all countries [3], since it was stated as a pandemic [4]. COVID-19 is a disease caused by severe acute respiratory syndrome coronavirus 2 (SARS-CoV-2), which is a worldwide public emergency [5]. COVID-19 pandemic has become one of the central health crises of a generation. This pandemic has affected all persons globally [6].

COVID-19 puts a significant burden comprising morbidity and mortality [7, 8]. It has also led to substantial economic disasters besides mortality and morbidity [9]. This pandemic has also led to mental health worsening of the families who had children [10], the entire population [11], and an enormous effect on mental health of the youth [12]. It has also affected the development of children [13] and interrupted the vaccination of children [14]. Furthermore, this pandemic also has momentous stress on patients, healthcare systems, and HCWs [15]. It has also affected the treatment and prevention of chronic cases such as *tuberculosis* and human immunodeficiency virus [16]. COVID-19 had put an extensive problem on the African continent [17], a poor and susceptible population [9].

Thus, urgent measures across all countries have been necessitated because of the substantial morbidity and socioeconomics of this pandemic [18]. Because of a lack of vaccine, diverse prevention approaches were executed. Testing, contact tracing, and social restrictions are among the most powerful approaches adopted globally [19]. For instance, several measures are being implemented by the African countries, including school closures, travel bans, limits to large gatherings, increased testing, and country lockdown [9].

In high-income countries or regions, COVID-19 vaccine hesitancy remains a highly prevalent problem. Being younger, females, non-Whites, and having lower education or income level were more prone to vaccine hesitancy. Furthermore, factors associated with vaccine hesitancy were history of not receiving influenza vaccination, a lower self-perceived risk of contracting COVID-19, a lesser fear for health outcomes or COVID-19, not believing in the severity of COVID-19, having concerns about the rapid development of COVID-19 vaccines as well as disbeliefs in the safety and effectiveness of the vaccines [20].

A vaccine offers the greatest hope for a permanent solution to control it [2]. Since COVID-19 is continuing its impact all over the country, the government should be equipped to distribute a COVID-19 vaccine accordingly [21]. The intention for a vaccine against COVID-19 is determined by the information concerning to the people variety, vaccine efficacy, and vaccine development [22]. Since there are controversies regarding the safety and efficacy of this vaccine, this may decline the vaccination rates [23]. Vaccine hesitancy may lead to the decrement in the need of the population for a COVID-19 vaccine [24]. Besides, people unwillingness for this vaccine will determine the COVID-19 response and public health benefits of an effective vaccine [25]. The effectiveness of COVID-19 vaccine will be tested by vaccine hesitancy [26]. Only a small proportion of the parents had agreed to vaccinate their children against COVID-19 [27]. About one-third of the caregivers were reluctant to vaccinate their children [28]. The parents are not agreed to join their child, even in a clinical trial for this vaccine [29]. This would delay the time of the pandemic, because all these factors affect the attainment of herd immunity to this pandemic [30].

Knowing the intention of this vaccine will assist in the application of effective methods to improve this vaccination [31]. Lessening vaccination hesitancy concerning COVID-19 to control it may be as notable as determining a safe and effective vaccine [32]. It is an ethical and humanistic responsibility to approve that this vaccine is safe for the public [33]. It is vital to permit HCWs and the community to have access to reliable and satisfactory evidence about this vaccine to increase its acceptance rate [34]. The attitude of the HCWs regarding COVID-19 vaccine affects themselves to use the vaccine and their willingness to recommend for the patients. Therefore, future education should prioritize for HCWs to the population to accept it [35]. HCWs who refuse to have vaccination are often accused of exposing their patients to a lethal infection [36]. It is acceptable that vaccines are very significant population health measures to defend individuals from this pandemic. Besides, HCWs accounted for a considerable figure of infected individuals [37].

The development of SARS-CoV-2 virus vaccine puts in itself a new test for governments and health authorities [38]. HCWs are at high risk of COVID-19 [39, 40]. The pandemic among these populations is a main worry for health authorities worldwide. While COVID-19 infection in HCWs would have an instant consequence on their occupation and the whole healthcare system [39]. Protecting the HCWs from COVID-19 would be critical to preserve healthcare systems [37]. A vaccine must be acknowledged and used by the population to be effective [2]. Developing trust between communities and the intention to take COVID-19 vaccine is as significant as producing a safe and effective vaccine to control this pandemic [41]. The study revealed that the decrease in COVID-19 cases among HCWs started after anti-COVID-19 vaccination, which reveals that COVID-19 vaccines are effective in preventing infection [42].

### 1.1. Research Questions

1. What is the global pooled prevalence of positive pro-vaccination attitude towards COVID-19 vaccine among HCWs and non-HCWs?
2. What are the factors associated with the level of positive pro-vaccination attitude towards COVID-19 vaccine among HCWs and non-HCWs?

## 2. Methods

### 2.1. Study Setting

Studies done across worldwide were included in to this systematic review and meta-analysis.

### 2.2. Search Strategies

Different databases such as PubMed, EMBASE, Scopus, Web of Science, and Google Scholar were used to search the related articles. During this, the search was done for articles published until August 31, 2022. Search terms used were; COVID-19, SARS-CoV-2, novel coronavirus, nCoV, severe acute respiratory syndrome coronavirus 2, coronavirus disease 2019 virus, 2019-nCoV, 2019 novel coronavirus, coronavirus, attitude, factors, associated factors, healthcare workers, nurses, midwives, physician, health professional, healthcare providers, and vaccine. Boolean operators' strings were used (Table 1).

## 3. Eligibility Criteria

### 3.1. Inclusion Criteria

Studies were included into the systematic review and meta-analysis if they fulfil: cross-sectional studies which reported outcome variables, articles done among adults, and articles published in English language, and articles published up to August 31, 2022, across all countries.

### 3.2. Exclusion Criteria

Articles which did not assess the outcome variables, articles which were not fully accessible, articles published in non-English language, and articles with poor quality were excluded from this systematic review and meta-analysis.

### 3.3. Outcome Interest

In this systematic review, the primary outcome was the prevalence of pro-vaccination attitude towards COVID-19 vaccine among HCWs and non-HCWs. Pro-vaccination attitude refers to the attitudes of the participants regarding to the COVID-19 vaccine, whether or not they take it if available. Pro-vaccination attitude was measured by using an “Yes” or “No” question. “Do you intend to have a COVID-19 vaccine in the future?” was the question asked to the participants. The secondary outcome was factors associated with pro-vaccination attitude towards COVID-19 vaccine among HCWs and non-HCWs which was reported within the included studies.

### 3.4. Data Extraction and Quality Assessment

The retrieved articles from all databases were exported to Thomson Reuters EndNote version 8. The titles and abstract of all possible articles to be included in this systematic review were checked. The standardized data extraction format prepared in a Microsoft Excel worksheet was used to extract the data from the selected articles according to the preset inclusion criteria. The names of the authors, publication year, study period, study country, participants, sample size, study design, prevalence, and factors were used for the extraction of data from each article.

This systematic review has only included cross-sectional studies. The NOS quality assessment criteria for cross-sectional studies were used to assess the included articles [43, 44], and the modified NOS for cross-sectional studies was used to include the articles. Whereas, all articles with ≥5 out of 10 were considered as a high-quality score [45], and included in to this systematic review and given as (supplementary 12 file). The NOS methodological quality assessment score has been included for each article (Table 2).

### 3.5. Data Processing and Analysis

Random effect model was used to estimate the global pooled prevalence of pro-vaccination attitude towards COVID-19 vaccine among both HCWs and non-HCWs. The analysis was done by using STATA version 11 statistical software. The heterogeneity of the included articles was assessed by using *I*^2^ statistics. The publication bias was measured by using Egger's test. Subgroup analysis was done based on the study participant, publication year, and sample size. Forest plot was used to show the pooled prevalence with 95%Cl.

### 3.6. Data Synthesis and Reporting

This systematic review and meta-analysis were conducted on global pro-vaccination attitude and associated factors towards COVID-19 vaccine among HCWs and non-HCWs. During this, PRISMA 2020 flowchart diagram [96], and PRISMA 2020 checklist [96] were used for the study screening, selection, and inclusion in to this systematic review. PRISMA 2020 checklist is given as (supplementary 2 file).

## 4. Results

### 4.1. Search Results

**Figure 1:**
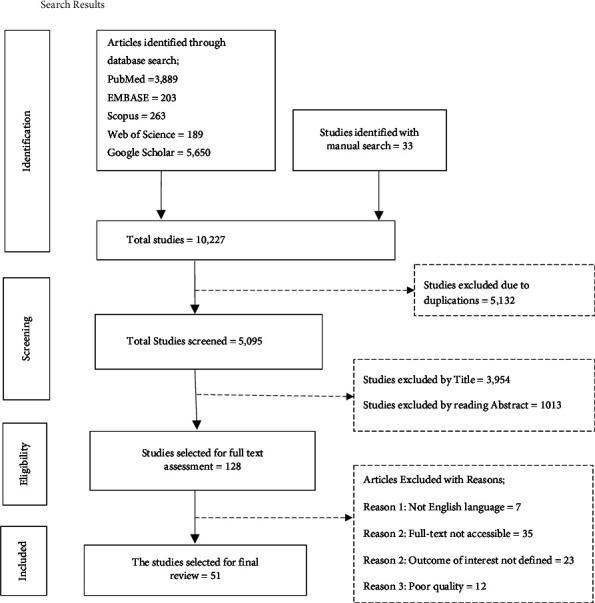
PRISMA flowchart diagram of the study selection for systematic review on pro-vaccination attitude and associated factors towards COVID-19 vaccine among HCWs and Non-HCWs globally. Note: adopted from Page MJ, McKenzie JE, Bossuyt PM, Boutron I Hoffmann TC, Mulrow CD, et al. The PRISMA 2020 statement: an updated guideline for reporting systematic reviews. BMJ. 2021; 372:n71. doi: 10.1136/bmj.n71. Reference [96].

All related studies done across the world were identified by using diverse databases. From the search made through the mentioned databases, 10,227 studies were found. From this, only 51 studies were meeting the predefined eligibility criteria and included in to this systematic review and meta-analysis (Figure 1).

### 4.2. Study Characteristics

This systematic review focused on the studies conducted on attitude regarding COVID-19 vaccine and its associated factors among the two major population categories, HCWs and non-HCWs. In this systematic review, a total of 48 studies were included, comprising of studies done on both HCWs and non-HCWs participants. There have been substantial differences concerning the level of attitude towards COVID-19 vaccine among both populations (Table 2).

### 4.3. Attitude towards COVID-19 Vaccine among HCWs

From the total of 51 studies included in to this systematic review and meta-analysis, only 23 studies were conducted among HCWs. The smallest and the largest sample sizes were reported from Germany (200) [61], and the United States (US) (5,287) [64], respectively. The smallest prevalence of a positive attitude towards COVID-19 vaccine among HCWs were reported as 21% from Egypt [60], while the largest prevalence was 95%, which was reported from Asia-Pacific [52]. Thus, the prevalence of a positive attitude towards COVID-19 vaccine among HCWs ranged from 21% [60] to 95% [52].

Factors significantly associated with the pro-vaccination attitude towards COVID-19 vaccine among HCWs were age, gender, race, work experience, home location, having no fear of injections, being a non-smoker, profession, presence of chronic illnesses, allergies, confidence in pharmaceutical companies, confidence in the management of the epidemic, history of taking influenza vaccine, vaccine recommendation, perceived risk of new vaccines, perceived utility of vaccine, receiving a seasonal flu vaccination in the last 5 years, working in a private hospital, a high perceived pandemic risk index, low vaccine harm index, high pro-socialness index, using Facebook as main information source about antiSARS-CoV-2 vaccination, being in close contact with a high-risk group, having undertaken seasonal flu vaccine during the 2019–2020 season, role within the hospital, knowledge about the virus, confidence in and expectations about personal protective equipment, and behaviors (Table 2).

### 4.4. Attitude towards COVID-19 Vaccine among Non-HCWs

Concerning to the non-HCWs, a total of 28 studies were conducted among non-HCWs from 48 studies included in to this systematic review. The smallest and the largest sample sizes were reported, 90 from the United States of America [90], and 32,361 from the United Kingdom [77], respectively. The smallest prevalence of a positive attitude towards COVID-19 vaccine among non-HCWs were reported as 21.4% from Lebanon [69], while the largest prevalence was 91.99%, which was reported from Poland [70]. Thus, the prevalence of a positive attitude towards COVID-19 vaccine among non-HCWs ranged from 21.4% [69] to 91.99% [70].

Factors associated with the attitude towards COVID-19 vaccine among non-HCWs were age, gender, educational level, occupation, marital status, residency, income, ethnicity, risk for severe course of COVID-19, direct contact with COVID-19 at work, being a health profession, being vaccinated against seasonal flu, perceived benefits, cues to actions, having previous history of vaccination, fear of passing on the disease to relatives, and the year of medical study, studying health-related courses, COVID-19 concern, adherence level to social distancing guidelines, history of chronic disease, being pregnant, perceived vaccine safety, having a close acquaintance who did not experience a vaccine-related adverse reaction, having more information about vaccine effectiveness, mandatory vaccination, being recommended to be vaccinated, lack of the confidence in the healthcare system to control epidemic, heard about COVID-19 vaccines, belief in COVID-19 vaccines protection from COVID-19 infection, those who encouraged their family members and friends to get vaccinated (Table 2).

### 4.5. Heterogeneity and Publication Bias

**Figure 2:**
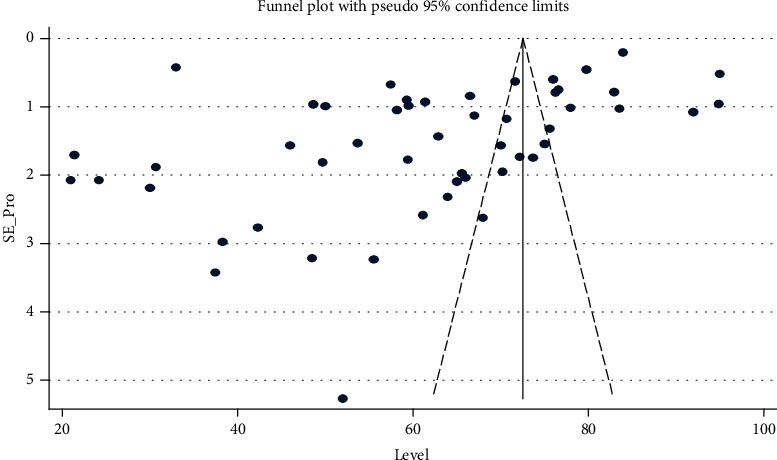
Funnel plot with 95% confidence limits of the pooled prevalence of pro-vaccination attitude towards COVID-19 vaccine among both HCWs and non-HCWs globally.

The heterogeneity and publication bias of the included studies in this meta-analysis were evaluated. There was a significant heterogeneity among the studies (*I*^2^ = 99.8%, *p*=0.000). The publication bias was determined by using Egger's test and the p-value was 0.003. Egger's test was statistically significant and the funnel plot showed the asymmetrical distribution of the included articles. Both of them suggest that there was publication bias (Figure 2).

### 4.6. Sensitivity Analysis

**Figure 3:**
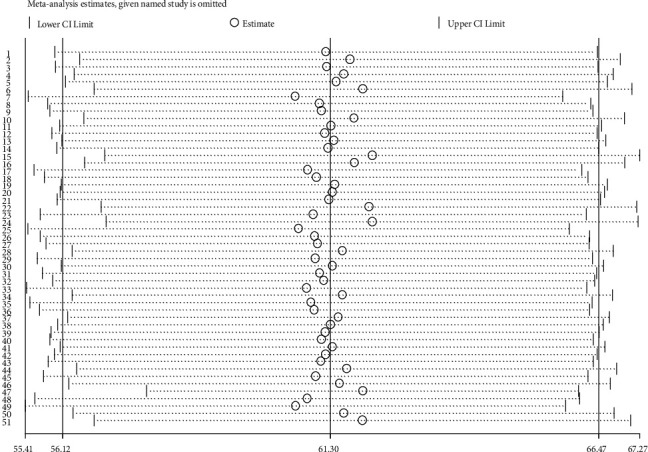
The results of sensitivity analysis of 51 studies conducted on pro-vaccination attitude towards COVID-19 vaccine among both HCWs and non-HCWs globally.

Using the random effects model, the results of a sensitivity analysis revealed that no single study influenced the overall prevalence of pro-vaccination attitude towards COVID-19 vaccine among both HCWs and non-HCWs globally (Figure 3).

### 4.7. Pro-Vaccination Attitude towards COVID-19 Vaccine

**Figure 4:**
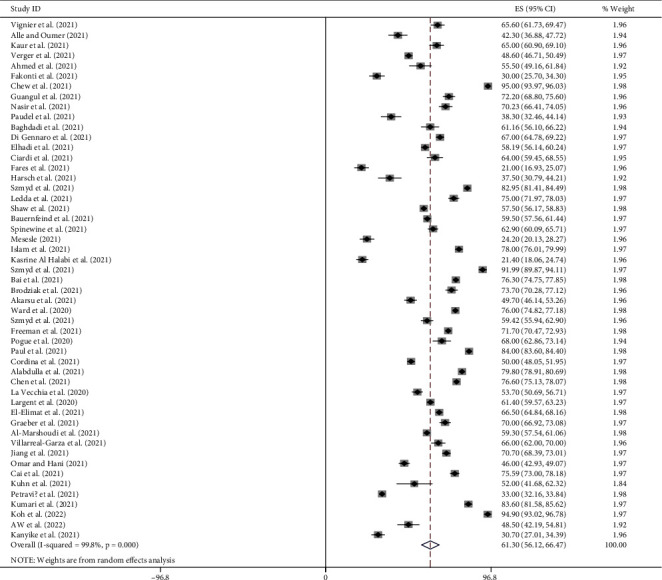
Forest plot of pooled prevalence of pro-vaccination attitude towards COVID-19 vaccine among both HCWs and non-HCWs globally.

Random effect model was used in this meta-analysis to estimate the global pooled prevalence of pro-vaccination attitude towards COVID-19 vaccine among both HCWs and non-HCWs. It was found to be 61.30% (95%CI: 56.12, 66.47). The level of heterogeneity was *I*^2^ = 99.8%: *p*=0.000 (Figure 4).

### 4.8. Subgroup Analysis

Due to the presence of a significant level of heterogeneity among the included studies, subgroup analysis was needed to identify the sources of heterogeneity. Therefore, subgroup analysis was done by using study participants, publication year, and sample size to determine the pooled prevalence of pro-vaccination attitude towards COVID-19 vaccine among both HCWs and non-HCWs globally.

### 4.9. Subgroup Analysis through the Study Participants

**Figure 5:**
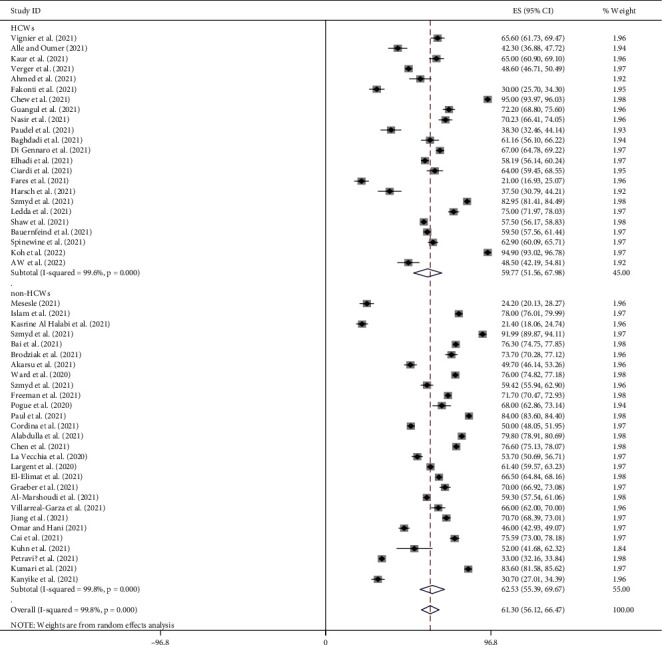
Subgroup analysis through study participants on the pooled prevalence of pro-vaccination attitude towards COVID-19 vaccine among both HCWs and non-HCWs globally.

The global pooled prevalence of pro-vaccination attitude towards COVID-19 vaccine was 59.77% (95%CI [51.56, 67.98]; *I*^2^ = 99.6%, *p*=0.000) among HCWs participants, and 62.53% (95%CI [55.39, 69.67]; *I*^2^ = 99.8%, *p*=0.000) among non-HCWs participants (Figure 5).

### 4.10. Subgroup Analysis by Sample Size

**Figure 6:**
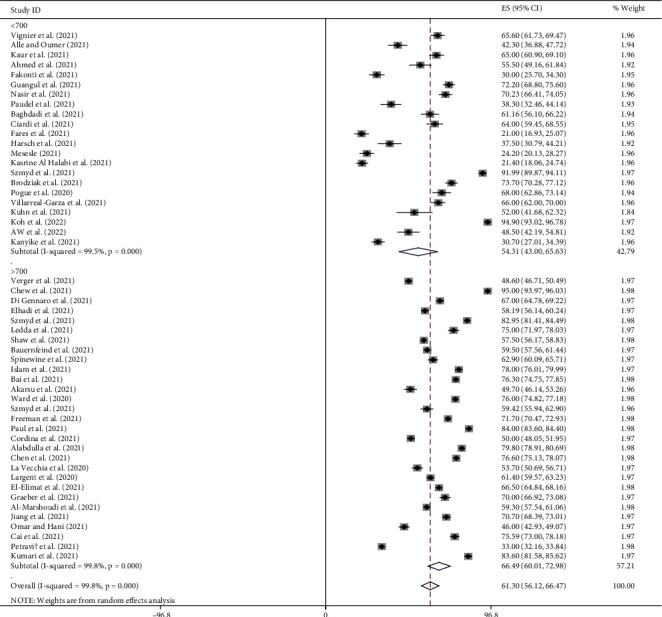
Subgroup analysis by sample size on the pooled prevalence of pro-vaccination attitude towards COVID-19 vaccine among both HCWs and non-HCWs globally.

The global pooled prevalence of pro-vaccination attitude towards COVID-19 vaccine among both HCWs and non-HCWs was 54.31% (95%CI [43, 65.63]; *I*^2^ = 99.5%, *p*=0.000) for sample size <700 and 66.49% (95%CI [60.01, 72.98]; *I*^2^ = 99.8%, *p*=0.000) for sample size >700 (Figure 6).

### 4.11. Subgroup Analysis by Year of Publication

**Figure 7:**
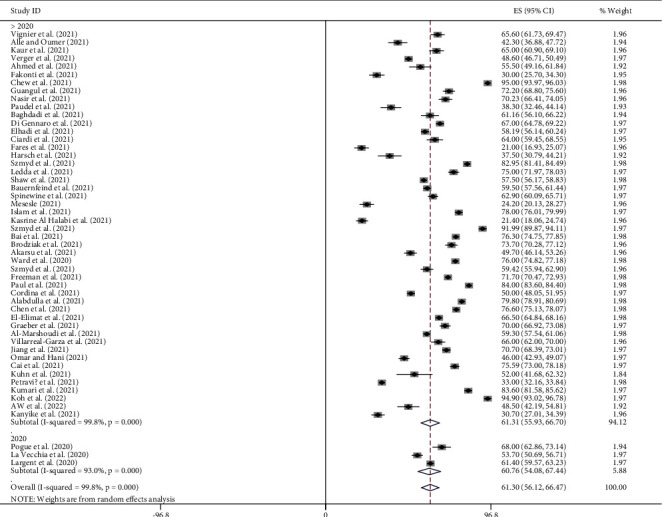
Subgroup analysis by year of publication on the pooled prevalence of pro-vaccination attitude towards COVID-19 vaccine among both HCWs and non-HCWs globally.

The global pooled prevalence of pro-vaccination attitude towards COVID-19 vaccine among both HCWs and non-HCWs was 60.70% (95%CI [54.08, 67.44]; *I*^2^ = 93.0%, *p*=0.000) for studies published in 2020 year and 61.31% (95%CI [55.93, 66.70]; *I*^2^ = 99.8%, *p*=0.000) for the studies published after 2020 years (Figure 7).

## 5. Discussion

Despite the fact that more than a year has passed since the WHO stated a COVID-19 pandemic, there is no effective treatment yet. The only strategy to halt the virus from spreading is the vaccination of the population as per the recent evidence. However, more populations should be vaccinated to achieve herd immunity. This is a substantial contest for healthcare systems. Having an effective vaccine is not equivalent to using it, public acceptance is crucial [97]. Besides, despite the consideration of vaccination good achievements of the twentieth century, there are remaining public health issues including insufficient, delayed, and unstable vaccination uptake [98]. Generally, the willingness to take the vaccine against COVID-19 will be the next main phase in fighting this pandemic. However, achieving high uptake will be a challenge and may be impeded by online misinformation and attaining significant uptake will be a contest [99].

Hence, this systematic review was intended to determine the pro-vaccination attitude and associated factors towards COVID-19 vaccine among HCWs and non-HCWs globally. Recognizing the level of attitude towards COVID-19 vaccine and its associated factors among concerning these two major populations would have a substantial role in managing and controlling this pandemic. This is due to the fact that this study provides critical evidences at the time of this global crisis, which is because of the adverse effects of the COVID-19 pandemic. This is supported by the study which explains that knowing the public needs and factors determining their attitude towards vaccines would assist to plan for multilevel interventions depending on the evidence to improve vaccine uptake globally [100]. Generally, to predict and be ready for the future epidemic and pandemic reply, it would be crucial to understand how populations approach emerging infectious diseases [101].

This systematic review and meta-analysis were done by using comprehensive search strategies. It was done based on PRISMA 2020 guidelines and checklists. The quality of the included studies was determined by using the modified NOS assessment. All included studies were cross-sectional. Publication bias was assessed by using Egger's test and funnel plots.

This meta-analysis revealed that the global pooled prevalence of pro-vaccination attitude towards COVID-19 vaccine among both HCWs and non-HCWs was 61.30% (95%CI: 56.12, 66.47, *I*^2^ = 99.8%: *p*=0.000). Due to the presence of a significant level of heterogeneity among the included studies, subgroup analysis was needed to identify the sources of heterogeneity. Therefore, subgroup analysis was done by using study participants, publication year, and sample size to determine the pooled prevalence of pro-vaccination attitude towards COVID-19 vaccine among both HCWs and non-HCWs globally. The global pooled prevalence of pro-vaccination attitude towards COVID-19 vaccine was 59.77% (95%CI [51.56, 67.98]; *I*^2^ = 99.6%, *p*=0.000) among HCWs participants, and 62.53% (95%CI [55.39, 69.67]; *I*^2^ = 99.8%, *p*=0.000) among non-HCWs participants. The global pooled prevalence of pro-vaccination attitude towards COVID-19 vaccine among both HCWs and non-HCWs was 54.31% (95%CI [43, 65.63]; *I*^2^ = 99.5%, *p*=0.000) for sample size <700 and 66.49% (95%CI [60.01, 72.98]; *I*^2^ = 99.8%, *p*=0.000) for sample size >700. The global pooled prevalence of pro-vaccination attitude towards COVID-19 vaccine among both HCWs and non-HCWs was 60.70% (95%CI [54.08, 67.44]; *I*^2^ = 93.0%, *p*=0.000) for studies published in 2020 year and 61.31% (95%CI [55.93, 66.70]; *I*^2^ = 99.8%, *p*=0.000) for the studies published after 2020 years.

The results of this systematic review showed that there was a substantial discrepancy on the level of attitude towards COVID-19 vaccine among HCWs and non-HCWs globally. The level of positive attitude towards COVID-19 vaccine among HCWs ranged from 21% [60] to 95% [52]. This finding demonstrates that there is a crucial problem that needs to be addressed on high priority to cease the era of the current pandemic. This is due to the fact that HCWs are at a high risk of COVID-19 [40]. This infection in HCWs would have an instant consequence on their occupation and the entire healthcare system [39]. Evidences revealed that greater than 50% of the global population have not been vaccinated. The vaccine coverage is less than 20% in some low- and middle-income countries [102]. The study conducted on acceptance of COVID-19 vaccination at different hypothetical efficacy and safety levels in ten countries in Asia, Africa, and South America revealed a higher possibility of side effects caused a large drop in COVID-19 vaccine acceptance rate at the same efficacy level. This showed the importance of accurate communication regarding vaccine safety and efficacy on attitude towards the vaccine and intentions to get vaccinated [103].

Factors associated with attitude towards COVID-19 vaccine among HCWs were age [47, 49, 50, 56, 57, 59], gender [50, 56, 59], race [59], work experience [56], home location [59], having no fear of injections [56], being a non-smoker [56], profession [47, 57], presence of chronic illnesses [50], allergy [50], confidence in pharmaceutical companies [46], confidence in the management of the epidemic [46], history of taking influenza vaccine [49], vaccine recommendation [49, 51], perceived risk of new vaccines [49], perceived utility of vaccine [49], receiving a seasonal flu vaccination in the last 5 years [51], working in a private hospital [51], a high perceived pandemic risk index [52], low vaccine harm index [52], high pro-socialness index [52], using Facebook as main information source about antiSARS-CoV-2 vaccination [57], being in close contact with a high-risk group [57], having undertaken seasonal flu vaccine during the 2019–2020 season [57], role within the hospital [59], knowledge about the virus [59], confidence in and expectations about personal protective equipment, and behaviors [59].

Concerning to non-HCWs, the level of positive attitude towards COVID-19 vaccine among non-HCWs was ranged from 21.4% [69] to 91.99% [70]. Factors associated with attitude towards COVID-19 vaccine among non-HCWs were age [65, 66, 74, 75, 86, 89], gender [65, 68, 69, 74, 75, 78, 85, 88], educational level [65, 88], occupation [65, 85], marital status [69, 88], residency [71, 88], income [74, 75], ethnicity [75], risk for severe course of COVID-19 [65], direct contact with COVID-19 at work [65], being a health profession [66, 78], being vaccinated against seasonal flu [65, 66, 88], perceived benefits [66], cues to actions [66], having previous history vaccination [68], fear of passing on the disease to relatives [70], and the year of medical study [70], studying health-related courses [71], COVID-19 concern [74], adherence level to social distancing guidelines [75], history of chronic disease [85], being pregnant [85], perceived vaccine safety [89], having a close acquaintance who did not experience a vaccine-related adverse reaction [86], having more information about vaccine effectiveness [86], mandatory vaccination [86], being recommended to be vaccinated [86], lack of the confidence in the healthcare system to control epidemic [88], heard about COVID-19 vaccines [89], believe in COVID-19 vaccines protection from COVID-19 infection [89], those who encouraged their family members and friends to get vaccinated [89].

Generally, the findings of this systematic review showed that several factors have been associated with the attitude towards COVID-19 vaccine among both HCWs and non-HCWs. This is because of the fact that even though the immunization coverage is stated administratively across the world, no likewise vigorous monitoring system occurs for vaccine confidence. There is rising evidence of vaccine denial because of the lack of trust in the benefits, safety, and effectiveness of vaccines [104]. The acceptance of a COVID-19 vaccine was vastly affected by the effectiveness of the vaccine [105]. Besides, if people lack enough knowledge towards the vaccine, this might lead to a negative attitude about it, which will avoid it to accept the vaccine. If communication efforts fail to address vaccine-negative persons', liberty-associated concerns may not be successful [106]. Even, the political talk was found to have a significant effect on the attitude of individuals. For instance, this study showed that political talk plays a considerable role in shaping and polarizing attitude on stem cell research [107]. The intention to accept this vaccine maybe affected by online misinformation, which is significantly associated with failure in vaccination intent [99]. Furthermore, vaccine-related conspiracy theories could affect the attitude of individuals towards the vaccine. This is supported by the experimental study conducted in China [108]. Moreover, according to the planned behavior theory, attitude regarding to behavior, subjective norms of behavior, and perceived control over behavior forecast behavioral willingness, while this willingness together with perceived behavioral control accounts for a substantial proportion of variance in behavior [109].

### 5.1. Limitations of the Study

It was difficult to consider some of the articles conducted on pro-vaccination attitude and associated factors towards COVID-19 vaccine among HCWs and non-HCWs, because they had not clearly measured the outcome variables. Moreover, some of those studies did not address factors associated with pro-vaccination attitude.

### 5.2. Recommendations

The acceptance of vaccines against COVID-19 is vital to fight this pandemic [110]. Hence, to rise the vaccination, considering the psychological science of action is suggested. It can be applied through: thoughts and feelings, social processes, and interventions, which can facilitate vaccination [98]. In the theory of normative conduct, norms have a substantial role in shaping human behavior. Thus, improving the probability of socially beneficial behavior in others via norm activation would be well advised [111]. Vaccination against COVID-19 pandemic might be a significant element of public health and fighting anti-vaccination attitude may assist this effort [112]. Preventing the attack on science, trust in scientists, and using nonconservative media for the better perception of COVID-19 vaccine is advised. The use of nonconservative media would rise the trust in scientists, whereas this would rise the certainty that COVID-19 vaccine could be a good solution for this pandemic. This is supported by the study conducted in the United States of America [113]. Considering the power and impact of media usage on social trust and risk perception, more efforts are required to confirm a correct and balanced information is being spread, while the social media in particular [114]. Social norms and family discussion might be fundamental in qualifying the community for the acceptance of COVID-19 vaccine. This is supported by the study done among Asian-Americans in the United States of America [115]. The coupled monitoring vaccine attitude and vaccination rates at the nationwide and subnational levels could support in identifying individuals with diminishing confidence and acceptance towards the vaccine [116]. Applying the protection motivation theory is also suggested for this pandemic. This is because, in the context of this theory, the individuals under threat made their protection decisions and coping judgements. According to the protection motivation theory, individuals under threat base their protection decisions on threat and coping appraisals. In the case of preventable communicable diseases, the theory holds that motivation for vaccination will be higher the more alarming a person's threat appraisals and the more promising her coping appraisals are [117]. Lastly, since rumors and conspiracy theories may bring mistrust which contributes to vaccine hesitancy, following the misinformation regarding a COVID-19 vaccine in real-time and using social media to distribute accurate information can support to protect the population from misinformation [118]. The campaigns and messaging concerning to take the vaccine against COVID-19 should consider the risk of COVID-19 to others and the requirement for everybody to take the vaccine [119]. Evolving communication to avoid vaccine hesitancy is significant to control COVID-19. Forwarding the effective messages to the public concerning this vaccine is crucial to promote the acceptance of this vaccine [120]. Campaigns to disseminate information are also vital to promote participation in the immunization of COVID-19 pandemic [121].

## 6. Conclusions

Despite the substantial crisis made by COVID-19 pandemic worldwide, it has not been managed and controlled effectively. The vaccines against COVID-19 have been developed, after a long wait and worldwide anxiety, as the best solution for this pandemic. The acceptance of vaccines against COVID-19 is vital to fight this pandemic. This meta-analysis revealed that the global estimated pooled prevalence of pro-vaccination attitude towards COVID-19 vaccine among both HCWs and non-HCWs was unsatisfactory. Whereas, according to this systematic review finding, the level of positive attitude towards COVID-19 vaccine among HCWs ranged from 21% to 95%. Age, gender, race, work experience, home location, having no fear of injections, being a non-smoker, presence of chronic illnesses, profession, allergies, confidence in pharmaceutical companies, confidence in the management of the epidemic, history of taking influenza vaccine, vaccine recommendation, perceived risk of new vaccines, perceived utility of vaccine, receiving a seasonal flu vaccination in the last 5 years, working in a private hospital, a high perceived pandemic risk index, low vaccine harm index, high pro-socialness index, using Facebook as main information source about antiSARS-CoV-2 vaccination, being in close contact with a high-risk group, having undertaken seasonal flu vaccine during the 2019–2020 season, role within the hospital, knowledge about the virus, confidence in and expectations about personal protective equipment, and behaviors were factors significantly associated with the attitude towards COVID-19 vaccine among HCWs.

The level of positive attitude towards COVID-19 vaccine among non-HCWs ranged from 21.4% to 91.99%. Factors associated with attitude towards COVID-19 vaccine among non-HCWs were age, gender, educational level, occupation, marital status, residency, income, ethnicity, risk for severe course of COVID-19, direct contact with COVID-19 at work, being a health profession, being vaccinated against seasonal flu, perceived benefits, cues to actions, having previous history of vaccination, fear of passing on the disease to relatives, and the year of medical study, studying health-related courses, COVID-19 concern, adherence level to social distancing guidelines, history of chronic disease, being pregnant, perceived vaccine safety, having a close acquaintance who did not experience a vaccine-related adverse reaction, having more information about vaccine effectiveness, mandatory vaccination, being recommended to be vaccinated, lack of the confidence in the healthcare system to control epidemic, heard about COVID-19 vaccines, believe in COVID-19 vaccines protection from COVID-19 infection, those who encouraged their family members and friends to get vaccinated.

The unfavorable attitude regarding COVID-19 vaccine among both HCWs and non-HCWs would significantly reduce the role of vaccination in dropping the burden of the COVID-19 pandemic throughout the community. Globally, there is a need for a call for action to cease the time and the associated crisis of this pandemic. This is because HCWs are the major source of health-related information for their communities. Thus, we need to equip them with the most truthful and reliable knowledge to improve their attitude towards COVID-19 vaccine.

## Figures and Tables

**Table 1:** Search databases and strategies about the HCWs and non-HCWs Pro-vaccination Attitude and Its Associated Factors Towards COVID-19 Vaccine.

Database	Search strategies
PubMed	(((“COVID-19”[All fields] OR “COVID-19”[MeSH terms] OR “COVID-19 vaccines”[All fields] OR “COVID-19 vaccines”[MeSH terms] OR “COVID-19 serotherapy”[All fields] OR “COVID-19 serotherapy”[Supplementary concept] OR “COVID-19 nucleic acid testing”[All fields] OR “COVID-19 nucleic acid testing”[MeSH terms] OR “COVID-19 serological testing”[All fields] OR “COVID-19 serological testing”[MeSH terms] OR “COVID-19 testing”[All fields] OR “COVID-19 testing”[MeSH terms] OR “SARS CoV 2”[All fields] OR “SARS CoV 2”[MeSH terms] OR “severe acute respiratory syndrome coronavirus 2”[All fields] OR “nCoV”[All fields] OR “2019 nCoV”[All fields] OR ((“coronavirus”[MeSH terms] OR “coronavirus”[All fields] OR “CoV”[All fields]) AND 2019/11/01 : 3000/12/31[date - publication])) AND (“attitude”[MeSH terms] OR “attitude”[All fields] OR “attitudes”[All fields] OR “attitude s”[All fields])) OR (“factor”[All fields] OR “factor s”[All fields] OR “factors”[All fields])) AND (“health personnel”[MeSH terms] OR (“health”[All fields] AND “personnel”[All fields]) OR “health personnel”[All fields] OR (“healthcare”[All fields] AND “workers”[All fields]) OR “healthcare workers”[All fields]) AND (“vaccine”[Supplementary concept] OR “vaccine”[All fields] OR “vaccination”[MeSH terms] OR “vaccination”[All fields] OR “vaccinable”[All fields] OR “vaccinal”[All fields] OR “vaccinate”[All fields] OR “vaccinated”[All fields] OR “vaccinates”[All fields] OR “vaccinating”[All fields] OR “vaccinations”[All fields] OR “vaccination s”[All fields] OR “vaccinator”[All fields] OR “vaccinators”[All fields] OR “vaccine s”[All fields] OR “vaccinated”[All fields] OR “vaccines”[MeSH terms] OR “vaccines”[All fields] OR “vaccine”[All fields] OR “vaccines”[All fields])

EMBASE	“COVID-19” OR “SARS-CoV-2” OR “novel coronavirus” OR “nCoV” OR “severe acute respiratory syndrome coronavirus 2” OR “coronavirus disease 2019 virus” OR “2019-nCoV” OR “2019 novel coronavirus” OR “severe acute respiratory syndrome coronavirus 2” OR “coronavirus AND “attitude” OR “factors” OR “associated factors” AND “healthcare workers” OR “nurses” OR “midwifes” OR “physician” OR “health professional” OR “healthcare providers” AND “vaccine”

Scopus	“COVID-19^*∗*^” OR “SARS-CoV-2^*∗*^” OR “novel coronavirus^*∗*^” OR “nCoV^*∗*^” OR “severe acute respiratory syndrome coronavirus 2^*∗*^” OR “coronavirus disease 2019 virus^*∗*^” OR “2019-nCoV^*∗*^” OR “2019 novel coronavirus^*∗*^” OR “coronavirus^*∗*^” AND “attitude^*∗*^” OR “factors^*∗*^” OR “associated factors^*∗*^” AND “healthcare workers^*∗*^” OR “nurses^*∗*^” OR “midwifes^*∗*^” OR “physician^*∗*^” OR “health professional^*∗*^” OR “healthcare providers^*∗*^” AND “vaccine^*∗*^”

Web of Science	(COVID-19 OR SARS-CoV-2 OR novel coronavirus OR nCoV OR severe acute respiratory syndrome coronavirus 2 OR coronavirus disease 2019 virus OR 2019-nCoV OR 2019 novel coronavirus OR severe acute respiratory syndrome coronavirus 2 OR coronavirus) AND attitude AND factors OR associated factors AND (healthcare workers OR nurses OR midwives OR physician OR health professional OR healthcare providers) AND vaccine

Google Scholar	COVID-19 OR SARS-CoV-2 OR novel coronavirus OR nCoV OR severe acute respiratory syndrome coronavirus 2 OR coronavirus disease 2019 virus OR 2019-nCoV OR 2019 novel coronavirus OR coronavirus AND attitude AND factors OR associated factors AND healthcare workers OR nurses OR midwives OR physician OR health professional OR healthcare providers AND vaccine

**Table 2:** Characteristics of the studies included in the systematic review and meta-analysis on the level of positive attitude towards COVID-19 vaccine among HCWs and non-HCWs over different countries.

S. N	Authors	Year	SP	Country	Participants	SS	SD	Level (%)	Factors	Quality score
1.	Vignier et al. [46]	2021	January 22 to March 26, 2021	France	HCWs	579	CS	65.6	Confidence in pharmaceutical companies, and confidence in the management of the epidemic.	8

2.	Alle and Oumer [47]	2021	February 5 to March 20, 2021	Ethiopia	Health professions	319	CS	42.3	Age and profession.	7

3.	Kaur et al. [48]	2021	Not explained	India	Medical and dental professionals	520	CS	65	NA	6

4.	Verger et al. [49]	2021	October and November 2020	France and French-speaking parts of Belgium and Canada	HCWs	2,678	CS	48.6	Age, history of taking influenza vaccine, vaccine recommendation, perceived risk of new vaccines, and perceived utility of vaccine.	7

5.	Ahmed et al. [50]	2021	Not explained	Saudi Arabia	Healthcare providers	236	CS	55.5	Sex, age, presence of chronic illnesses, and allergy.	8

6.	Fakonti et al. [51]	2021	December 8 to 28, 2020	Cyprus	Nurses and midwives	437	CS	30	Receiving a seasonal flu vaccination in the last 5 years, recommended vaccines for health professionals, and working in a private hospital.	7

7.	Chew et al. [52]	2021	December 12 to 21, 2020	Asia-Pacific	HCWs	1720	CS	95	A high perceived pandemic risk index, low vaccine harm index and high pro-socialness index.	8

8.	Guangul et al. [53]	2021	Not explained	Ethiopia	HCWs	668	CS	72.2	NA	6

9.	Nasir et al. [54]	2021	In February 2021	Bangladesh	HCWs	550	CS	70.23	NA	6

10.	Paudel et al. [55]	2021	January 27 to February 3, 2021.	Nepal	HCWs	266	CS	38.3	NA	6

11.	Baghdadi et al. [56]	2021	July to September 2020	Saudi Arabia	HCWs	356	CS	61.16	Gender, age (middle aged), work experience (<5 years), having no fear of injections, and being a non-smoker.	8

12.	Di Gennaro et al. [57]	2021	1 October to 1 November 2020	Italy	HCWs	1723	CS	67	Being a non-MD health professional, using Facebook as main information source about antiSARS-CoV-2 vaccination, being a younger, age (<30 years), being in close contact with a high-risk group, and having undertaken seasonal flu vaccine during the 2019–2020 season.	8

13.	Elhadi et al. [58]	2021	December 1 to 18, 2020	Libya	Physicians and paramedic	2215	CS	58.19	NA	8

14.	Ciardi et al. [59]	2021	December 10, 2020 to January 5, 2021	New York	HCWs	428	CS	64	Gender, age, race, home location, role within the hospital, knowledge about the virus, and confidence in and expectations about personal protective equipment and behaviors.	7

15.	Fares et al. [60]	2021	December 2020 to January 2021	Egypt	HCWs	385	CS	21	NA	6

16.	Harsch et al. [61]	2021	Not explained	Germany	HCWs	200	CS	37.5	NA	6

17.	Szmyd et al. [62]	2021	December 22, 2020 to January 8, 2021	Poland	HCWs	2300	CS	82.95	NA	6

18.	Ledda et al. [63]	2021	September to December 20, 2020	Italy	Healthcare personnel	787	CS	75	NA	6

19.	Shaw et al. [64]	2021	November 23 to December 5, 2020	US	Healthcare personnel	5287	CS	57.5	NA	6

20.	Bauernfeind et al. [65]	2021	December 12 to 21, 2020	Germany	Hospital employees	2454	CS	59.5	Age, gender, educational level, risk for severe course of COVID-19, occupation, direct contact with COVID-19 at work, flu shot in influenza 2019/2020, and flu shot in influenza 2020/2021.	8

21.	Spinewine et al. [66]	2021	January 6 to 20, 2021	Belgium	Hospital staffs	1132	CS	62.9	Being older, being a physician, being vaccinated against seasonal flu, perceived benefits, and cues to actions.	8

22.	Mesesle [67]	2021	March 13 to April 10, 2021	Ethiopia	Adult population	425	CS	24.2	NA	8

23.	Islam et al. [68]	2021	December 2020 to February 2021	Bangladesh	Adult population	1658	CS	78	Being female, and having previous history vaccination.	6

24.	Kasrine Al Halabi et al. [69]	2021	November to December 2020	Lebanon	Adult population	579	CS	21.4	Gender and marital status.	8

25.	Szmyd et al. [70]	2021	December 22 to 25, 2020	Poland	Medical students	632	CS	91.99	Fear of passing on the disease to relatives, and the year of medical study.	7

26.	Bai et al. [71]	2021	December 27, 2020 to January 18, 2021	China	College students	2,881	CS	76.3	Residency (urban), and studying health-related courses.	7

27.	Brodziak et al. [72]	2021	Not explained	Poland	*Cancer* patients	635	CS	73.7	NA	8

28.	Akarsu et al. [73]	2021	10/06/2020 and 10/07/2020	Turkey	Adult population	759	CS	49.7	NA	6

29.	Ward et al. [74]	2020	Each week of April 2020	France	Adult population	5018	CS	76	Gender, age, COVID-19 concern, and HICU.	8

30.	Szmyd et al. [70]	2021	December 22 to 25, 2020	Poland	Nonmedical students	763	CS	59.42	NA	8

31.	Freeman et al. [75]	2021	September 24 to October 17, 2020	UK	Adult population	5,114	CS	71.7	Younger age, female gender, lower income, ethnicity, and lower adherence to social distancing guidelines.	7

32.	Pogue et al. [76]	2020	Not explained	United States	Adult population	316	CS	68	NA	6

33.	Paul et al. [77]	2021	March 21/2020	UK	Adult population	32,361	CS	84	NA	8

34.	Cordina et al. [78]	2021	30/10/2020 to 16/11/2020	Malta	Adult population	2529	CS	50	Gender(male), and being health profession.	7

35.	Alabdulla et al. [79]	2021	October 15 to November 15, 2020	Qatar	Adult population	7821	CS	79.8	NA	8

36.	Chen et al. [80]	2021	Not explained	China	Adult population	3195	CS	76.6	NA	7

37.	La Vecchia et al. [81]	2020	September 16 to 28, 2020	Italy	15–85 years population	1055	CS	53.7	NA	6

38.	Largent et al. [82]	2020	September 14 to 27, 2020	US	Adult population	2730	CS	61.4	NA	6

39.	El-Elimat et al. [83]	2021	November 2020	Jordan	Adult population	3,100	CS	66.5	NA	8

40.	Graeber et al. [84]	2021	June and July 2020	Germany	Adult population	851	CS	70	NA	7

41.	Al-Marshoudi et al. [85]	2021	December15 to 31, 2020	Oman	Adult population	3000	CS	59.3	Gender (male), history of chronic disease, pregnancy, perceived vaccine safety, education levels, and occupation.	8

42.	Villarreal-Garza et al. [86]	2021	March 12 to 26, 2021	Mexico	Breast cancer patients	540	CS	66	Age, having a close acquaintance who did not experience a vaccine-related adverse reaction, having more information about vaccine effectiveness, mandatory vaccination, and being recommended by their oncologist to be vaccinated.	6

43.	Jiang et al. [87]	2021	Mid-March 2021	China	Nursing college students	1,488	CS	70.07	NA	6

44.	Omar and Hani [88]	2021	January 7 to March 30, 2021	Egypt	Adult population	1011	CS	46	Gender (female), residence (urban), educational level (university/post graduate), marital status (married), having flu vaccine, and lack of the confidence in the healthcare system to control epidemic.	7

45.	Cai et al. [89]	2021	November 27, 2020 and March 12, 2021	China	Adolescent population	1,057	CS	75.59	Age (younger), heard about COVID-19 vaccines, believe in COVID-19 vaccines protection from COVID-19 infection, and those who encouraged their family members and friends to get vaccinated, and believing that vaccines are safe.	8

46.	Kuhn et al. [90]	2021	December 2020 to January 2021	USA	PEH	90	CS	52	NA	7

47.	Petravić et al. [91]	2021	December 17 to 27, 2020	Slovenia	Residents >15 years	12,042	CS	33	NA	8

48.	Kumari et al. [92]	2021	March 13 to 25, 2021	India	≥18 years population	1294	CS	83.6	NA	7

49.	Koh et al. [93]	2022	May to June							

2021	Singapore	Primary healthcare workers	528	CS	94.9%	NA	7			

50.	AW et al. [94]	2022	March to July 2021	Singapore	HCWs	241	CS	48.5	Being female, a younger age, not having had a loved one or friend infected with COVID-19 and obtaining information from newspapers	7

51.	Kanyike et al. [95]	2021	March 15 to 21 2021	Uganda	Medical students	600	CS	30.7	NA	7

Notice: SP, study period; SS, sample size; SD, study design; CS, cross-sectional; HCWs, healthcare workers; NA, not applicable; HICU, household income per consumption unit; PEH, people experiencing homelessness.

## Data Availability

The data used to support the findings of this study are included within the article.

## Abbreviations

COVID-19: Coronavirus disease 2019
HCWs: Healthcare workers
non-HCWs: Nonhealthcare worker
PRISMA: Preferred Reporting Items for Systematic reviews and Meta-Analyses
US: the United States
NOS: Newcastle-Ottawa Scale.

## Supplementary Materials

Supplementary Materials Supplementary 1 file: Methodological quality assessment of cross-sectional studies using modified Newcastle-Ottawa Scale (NOS). Supplementary 2 file: PRISMA 2020 Checklist.
