# Supplementary material for: Healthcare Workers and Nonhealthcare Workers Pro-Vaccination Attitude and Its Associated Factors towards COVID-19 Vaccine Globally: A Systematic Review and Meta-Analysis
Source: Interdiscip Perspect Infect Dis. 2022 Oct 10;2022:2443785. doi: 10.1155/2022/2443785 (PMC9576430; doi:10.1155/2022/2443785)
Supplement: Supplementary Materials — Supplementary 1 file: Methodological quality assessment of cross-sectional studies using modified Newcastle-Ottawa Scale (NOS). Supplementary 2 file: PRISMA 2020 Checklist. [file 2443785.f1.zip › Supplementary 1 file.docx]

**Methodological Quality Assessment**

Supplementary file 1: Methodological quality assessment of cross-sectional studies using modified Newcastle - Ottawa Scale (NOS)

| **S. N** | **First author** | **publication year** | **Criteria** | | | | | | | | |
| --- | --- | --- | --- | --- | --- | --- | --- | --- | --- | --- | --- |
|  |  |  | **Selection** | | | | **Comparability** | | **Outcome** | | **Total score** |
|  |  |  | **Representativeness of the sample** | **Sample size** | **Non –responders** | **Ascertainment of exposure/risk factor** | **The study controls for the most important factor** | **The study control for any additional factor** | **Assessment of the outcome** | **Statistical test** |  |
|  | Vignier et al. | 2021 | **B*** | **A*** | **B*** | **A*** | **A*** | **B*** | **A*** | **A*** | **8** |
|  | Alle and Oumer | 2021 | **A*** | **A*** | **A*** | **A*** | **A*** | **-** | **A*** | **A*** | **7** |
|  | Kaur et al. | 2021 | **B*** | **A*** | **B*** | **A*** | **-** | **-** | **A*** | **A*** | **6** |
|  | Verger et al. | 2021 | **A*** | **A*** | **-** | **A*** | **A*** | **A*** | **A*** | **A*** | **7** |
|  | Ahmed et al. | 2021 | **A*** | **A*** | **B*** | **A*** | **A*** | **B*** | **A*** | **A*** | **8** |
|  | Fakonti et al. | 2021 | **A*** | **A*** | **-** | **A*** | **A*** | **A*** | **A*** | **A*** | **7** |
|  | Chew et al. | 2021 | **A*** | **A*** | **A*** | **A*** | **A*** | **A*** | **A*** | **A*** | **8** |
|  | Guangul et al. | 2021 | **A*** | **A*** | **B*** | **A*** | **-** | **-** | **A*** | **A*** | **6** |
|  | Nasir et al. | 2021 | **B*** | **A*** | **A*** | **A*** | **-** | **-** | **A*** | **A*** | **6** |
|  | Paudel et al. | 2021 | **A*** | **A*** | **B*** | **A*** | **-** | **-** | **A*** | **A*** | **6** |
|  | Baghdadi et al. | 2021 | **B*** | **A*** | **A*** | **A*** | **A*** | **A*** | **A*** | **A*** | **8** |
|  | Di Gennaro et al. | 2021 | **A*** | **A*** | **A*** | **B*** | **A*** | **B*** | **A*** | **A*** | **8** |
|  | Elhadi et al. | 2021 | **B*** | **A*** | **A*** | **A*** | **A*** | **A*** | **A*** | **A*** | **8** |
|  | Ciardi et al. | 2021 | **B*** | **A*** | **A*** | **A*** | **A*** | **-** | **A*** | **A*** | **7** |
|  | Fares et al. | 2021 | **A*** | **A*** | **A*** | **A*** | **-** | **-** | **A*** | **A*** | **6** |
|  | Harsch et al. | 2021 | **A*** | **A*** | **A*** | **B*** | **-** | **-** | **A*** | **A*** | **6** |
|  | Szmyd et al. | 2021 | **A*** | **A*** | **A*** | **A*** | **-** | **-** | **A*** | **A*** | **6** |
|  | Ledda et al. | 2021 | A* | **A*** | **A*** | **A*** | **-** | **-** | A* | **A*** | **6** |
|  | Shaw et al. | 2021 | **B*** | **A*** | **B*** | **A*** | **-** | **-** | **A*** | **A*** | **6** |
|  | Bauernfeind et al. | 2021 | B* | **A*** | **B*** | **A*** | **A*** | **B*** | A* | **A*** | **8** |
|  | Spinewine et al. | 2021 | **B*** | **A*** | **A*** | **A*** | **A*** | **A*** | **A*** | **A*** | **8** |
|  | Mesesle | 2021 | **B*** | **A*** | **A*** | **A*** | **A*** | **B*** | **A*** | **A*** | **8** |
|  | Islam et al | 2021 | B* | **A*** | **A*** | **A*** | **-** | **-** | A* | **A*** | **6** |
|  | Kasrine Al Halabi et al. | 2021 | **A*** | **A*** | **A*** | **A*** | **A*** | **A*** | **A*** | **A*** | **8** |
|  | Szmyd et al. | 2021 | **B*** | **A*** | **A*** | **A*** | **A*** | **-** | **A*** | **A*** | **7** |
|  | Bai et al. | 2021 | **B*** | **A*** | **A*** | **A*** | **A*** | **-** | **A*** | **A*** | **7** |
|  | Brodziak et al. | 2021 | **B*** | **A*** | **A*** | **A*** | **A*** | **A*** | **A*** | **A*** | **8** |
|  | Akarsu et al. | 2021 | **B*** | **A** | **A*** | **A*** | **-** | **-** | **A*** | **A*** | **6** |
|  | Ward et al. | 2020 | **A*** | **A*** | **A*** | **A*** | **A*** | **A*** | **A*** | **A*** | **8** |
|  | Szmyd et al. | 2021 | **B*** | **A*** | **A*** | **A*** | **A*** | **A*** | **A*** | **A*** | **8** |
|  | Freeman et al. | 2021 | **A*** | **A*** | **A*** | **A*** | **A*** | **-** | **A*** | **A*** | **7** |
|  | Pogue et al. | 2020 | **B*** | **A*** | **A*** | **A*** | **-** | **-** | **A*** | **A*** | **6** |
|  | Paul et al. | 2021 | **A*** | **A*** | **A*** | **A*** | **A*** | **A*** | A* | **A*** | **8** |
|  | Cordina et al. | 2021 | **A*** | **A*** | **A*** | **A*** | **A*** | **-** | **A*** | **A*** | **7** |
|  | Alabdulla et al. | 2021 | **A*** | **A*** | **A*** | **A*** | **A*** | **A*** | **A*** | **A*** | **8** |
|  | Chen et al. | 2021 | **A*** | **A*** | **A*** | **A*** | **A*** | **-** | **A*** | **A*** | **7** |
|  | La Vecchia et al. | 2020 | **A*** | **A*** | **A*** | **A*** | **-** | **-** | **A*** | **A*** | **6** |
|  | Largent et al. | 2020 | **A*** | **A*** | **A*** | **A*** | **-** | **-** | **A*** | **A*** | **6** |
|  | El-Elimat et al. | 2021 | **A*** | **A*** | **A*** | **A*** | **A*** | **A*** | **A*** | **A*** | **8** |
|  | Graeber et al. | 2021 | **A*** | **A*** | **A*** | **A*** | **A*** | **-** | **A*** | **A*** | **7** |
|  | Al-Marshoudi et al. | 2021 | **A*** | **A*** | **A*** | **A*** | **A*** | **A*** | **A*** | **A*** | **8** |
|  | Villarreal-Garza et al. | 2021 | **B*** | **A*** | **A*** | **-** | **A*** | **-** | **A*** | **A*** | **6** |
|  | Jiang et al. | 2021 | **B*** | **A*** | **A*** | **A*** | **-** | **-** | **A*** | **A*** | **6** |
|  | Omar and Hani | 2021 | **A*** | **A*** | **A*** | **A*** | **A*** | **-** | A* | **A*** | **7** |
|  | Cai et al. | 2021 | **A*** | **A*** | **A*** | **A*** | **A*** | **B*** | **A*** | **A*** | **8** |
|  | Kuhn et al. | 2021 | **A*** | **A*** | **A*** | **A*** | **A*** | **-** | **A*** | **A*** | **7** |
|  | Petravić et al. | 2021 | **A*** | **A*** | **A*** | **A*** | **A*** | **B*** | **A*** | **A*** | **8** |
|  | Kumari et al. | 2021 | **A*** | **A*** | **A*** | **A*** | **A*** | **-** | A* | **A*** | **7** |
|  | Koh et al. | 2022 | **A*** | **A*** | **A*** | **A*** | **A*** | **-** | A* | **A*** | **7** |
|  | AW et al. | 2022 | **B*** | **B*** | **B*** | **A*** | **A*** | **-** | A* | **A*** | **7** |
|  | Kanyike et al. | 2021 | **B*** | **B*** | **C*** | **A*** | **A*** | **-** | A* | **A*** | **7** |

*Note: from each item account point. (Accept the study if total score ≥5)*

Selection: (Maximum 5 stars)
1) Representativeness of the sample: a) Truly representative of the average in the target population. * (all subjects or random sampling) .b) Somewhat representative of the average in the target population. * (nonrandom sampling) .c) Selected group of users.d) No description of the sampling strategy.
2) Sample size:a) Justified and satisfactory. *.b) Not justified.
3) Non-respondents: a) Comparability between respondents and non-respondents characteristics is
established, and the response rate is satisfactory. * .b) The response rate is unsatisfactory, or the comparability between respondents
and non-respondents is unsatisfactory. c) No description of the response rate or the characteristics of the responders and
the non-responders.
4) Ascertainment of the exposure (risk factor): a) validated measurement tool. ** .b) Non-validated measurement tool, but the tool is available or described.* c) No description of the measurement tool.
Comparability: (Maximum 2 stars)
1) The subjects in different outcome groups are comparable, based on the study design or analysis. Confounding factors are controlled. a) The study controls for the most important factor (select one). * b) The study control for any additional factor. *
Outcome: (Maximum 3 stars)
1) Assessment of the outcome: a) Independent blind assessment. **,b) Record linkage. **,c) Self report. *,d) No description.
2) Statistical test:a) The statistical test used to analyze the data is clearly described and appropriate, and the measurement of the association is presented, including confidence intervals and the probability level (p value). *,b) The statistical test is not appropriate, not described or incomplete
